# Lifetime Abortion of Female Sex Workers in Iran: Findings of a National Bio-Behavioural Survey In 2010

**DOI:** 10.1371/journal.pone.0166042

**Published:** 2016-11-18

**Authors:** Mohammad Karamouzian, Ali Mirzazadeh, Mostafa Shokoohi, Razieh Khajehkazemi, Abbas Sedaghat, Ali Akbar Haghdoost, Hamid Sharifi

**Affiliations:** 1 HIV/STI Surveillance Research Center, and WHO Collaborating Center for HIV Surveillance, Institute for Futures Studies in Health, Kerman University of Medical Sciences, Kerman, Iran; 2 School of Population and Public Health, Faculty of Medicine, University of British Columbia, Vancouver, BC, Canada; 3 Department of Epidemiology and Biostatistics, University of California San Francisco, San Francisco, CA, United States of America; 4 Epidemiology & Biostatistics, Schulich School of Medicine & Dentistry, The University of Western Ontario, London, ON, Canada; 5 Modeling in Health Research Center, Institute for Futures Studies in Health, Kerman University of Medical Sciences, Kerman, Iran; 6 Center for Disease Control (CDC), Ministry of Health and Medical Education, Tehran, Iran; University of Washington, UNITED STATES

## Abstract

**Introduction:**

Unintended pregnancies and abortion may be considered as occupational hazards for female sex workers (FSWs). As our understanding of contraceptive and abortion practices of Iranian FSWs is very limited, this study tries to assess the dynamics of contraception and abortion among this sub-population.

**Methods:**

This survey was conducted in 2010, by recruiting 872 FSWs through facility-based sampling from 21 sites in 14 cities in Iran. Data were collected through face-to-face interviews using a pilot-tested standardized risk assessment questionnaire. We applied the logistic regression model to investigate the correlates of induced abortion among FSWs.

**Results:**

Of the 863 participants with valid responses to the abortion variable, 35.3% (95% CI: 32.1–38.6) acknowledged ever induced abortion and the annual rate of abortion was estimated at 20.7 per 1000 women. Around 31.2% of FSWs reported no usual contraceptive use, 32.6% barrier method, 23.6% non-barrier modern contraception methods, and 12.5% dual protection. In our multivariable model, older age (Adjusted Odds Ratio (AOR) = 1.74, 95% Confidence Interval (CI): 1.02, 2.96), group sex (AOR = 1.92, 95% CI: 1.10, 3.35), history of travel for sex work (AOR = 1.55, 95% CI: 1.09, 2.20), sexual violence (AOR = 1.77, 95% CI: 1.25, 2.50), STIs in last year (AOR = 1.53, 95% CI: 1.09, 2.14), and accessing family planning services (AOR = 1.76, 95% CI: 1.24, 2.49) were significant predictors of lifetime abortion.

**Conclusions:**

The reproductive health needs of Iranian FSWs are unmet and around one-third of FSWs reported induced abortion. Scaling-up comprehensive family planning services and empowering FSWs to have safer sex practices may help them to prevent unintended pregnancies and further risk of HIV transmission.

## Introduction

Female sex workers’ (FSWs) sexual and reproductive health needs are complicated given their vulnerability to sexually transmitted infections (STIs) and unintended pregnancies [1]. However, due to the sensitive nature of sex work, they remain underrepresented in fertility-related research and their fertility needs are often invisible or overshadowed by their ‘risky’ occupation [1]. Several studies have reported high HIV and STIs prevalence, physical and sexual violence, and limited access to healthcare services among FSWs; all of which have adverse effects on their pregnancy outcomes [1–4]. Not often recognized as mothers, health risks associated with pregnancy outcomes in FSWs remain overlooked [2, 5].

Recent research, however, suggests that despite complex reasons for pregnancy among FSWs, most desire to have or have children [3, 5, 6]. While some FSWs may consider having children as a way to solidify their relationship with a sex partner or leave sex work, others may be forced into sex work to provide for their children [1, 5, 7]. On the other hand, unintended pregnancies–as an occupational hazard–are very frequent among FSWs across different settings and often lead to abortion [1, 2]. Evidence suggests that many abortions among this population are unsafe, and most FSWs suffer from its complications [1, 8]. Unsafe practices of abortion among FSWs are mainly driven by the criminalization of sex work and abortion [1].

In the context of Iran, similar to several other settings, sex work remains criminalized and highly stigmatized [2, 4, 9]. Additionally, induced abortion is illegal, except for medical indications that are life-threatening to mother or diagnoses of congenital fetal defects (e.g., Down syndrome) [10, 11]. Despite the criminalization of induced abortion, unsafe abortions do take place underground and studies in Iran suggest an annual rate of around eight abortions per 1,000 women of reproductive ages [10, 11]. Iranian health policy makers have recently scaled up health care services, harm reduction efforts and educational interventions catered towards FSWs. However, criminalization of abortion and female sex work have created barriers in the recognition of important contraceptive needs of FSWs in Iran. Given the considerable burden of HIV among Iranian FSWs (i.e., Point prevalence of 4.5%) [12] and the intersection of HIV and reproductive health outcomes among FSWs through mother-to-child transmission of the virus, it is essential to inform policies aimed at improving FSWs’ reproductive health outcomes by characterizing abortion practices among Iranian FSWs. Using data from the first national survey of FSWs, we try to describe characteristics of lifetime abortion among FSWs in Iran.

## Materials and Methods

### Study design and data collection

The first Bio-Behavioural Surveillance Survey (BBSS) of Iranian FSWs was undertaken between April and July 2010. Details of methodology and sampling strategies of the survey are described elsewhere [12, 13]. Briefly, eligible participants were i) ≥18 years; ii) had exchanged sex for money, drugs, or goods at least once during the previous year; iii) had a history of practising sex work for at least 6 months; iv) held Iranian citizenship; v) were residents of the respective city; and vi) consented for participation. Data collection was anonymous, and no names or personal identifying information was recorded during recruitment, informed consent, or interview, or HIV testing. Through a pilot-tested risk assessment questionnaire, demographic and behavioural data was collected from 21 facilities catered towards vulnerable women (e.g., FSWs, partners of PWID, and women who inject drugs) located in 14 cities. Facilities were selected based on suspected level of HIV prevalence in FSWs and the local expert opinion of HIV surveillance departments on facilities’ logistics and capacity constraints. A convenience sample of 30–45 participants was recruited at each facility. Out of 1005 women initially approached, some participants were excluded for not meeting the eligibility criteria (n = 33) and the low quality of data collection in one province where our protocol for confidentiality and collection of data was not entirely followed (n = 100). Out of the 872 eligible women, nine did not answer to the question on lifetime abortion (final analytic sample = 863).

### Dependent variable: Lifetime abortion

The current analysis explores the correlates of lifetime abortion among FSWs through a self-reported history of ever having an abortion. Lifetime abortion was treated as a binary variable and the responses to the question “*Have you ever had an abortion*?” were coded as yes or no.

### Independent variables

Independent variables included age at interview (18–24, 25–34, or ≥35 years old), education (never attended school, ≤primary school, middle school, or ≥high school), ever married (yes or no), age at first marriage (≤18 or >18 years old), having other sources of income than sex work (yes or no), having children (yes or no), length of sex work (≤5 or >5 years), and HIV sero-status (positive or negative). Moreover, variables about sexual behaviour included age at first sex (≤18 or >18 years old), age at sex work debut (≤18 or >18 years old), multiple paying partners in last working day (yes or no), consistent condom use with paying partner (yes or no), consistent condom use with non-paying partner (yes or no), history of selling sex in brothels (yes or no), group sex in last week (yes or no), ever traveled for sex work (yes or no), experienced sexual violence in last month (yes or no), had STIs in last year (yes or no), accessed family planning services (yes or no), self-perceived risk of HIV (yes or no), ever tested for HIV (yes or no), usual contraceptive use (barrier (i.e., condom) or non-barrier modern contraception methods (i.e, Intra-Uterine Device, Implant, Sterilization, Oral contraceptives) or dual protection (i.e., concurrent condom and any non-barrier modern contraception method), or none). Lastly, substance-related variables included ever consumed alcohol (yes or no), ever used drugs (yes or no), and ever injected drugs (yes or no).

### Statistical analysis

Frequencies and descriptive statistics were computed for all variables. As FSWs were recruited from different facilities across the country, the recruitment sites were considered as sampling units, and their clustering effects were adjusted using Stata survey package. Annual rates of abortion among FSWs were estimated using the following formula:

1000* Number of lifetime abortions in the studied populationAverage number of reproductive years*Sample size

Bivariable and multivariable logistic regression models were constructed to investigate the correlates of lifetime abortion among FSWs. Crude and adjusted odds ratios (AORs), as well as 95% confidence intervals (CI), were reported. Variables with a P-value <0.2 in the bivariable analysis were entered into the multivariable regression model. Stata version 11 (Stata Corp.) was used throughout the analysis and P-values less than 0.05 were considered statistically significant.

### Ethical considerations

Ethical issues in this survey included the guarantee of the participants’ confidentiality and verbal informed consent for the behavioural interview and blood sample collection. Participants’ refusal to participate in the survey did not influence the services provided to them. The ethics committee of the Kerman University of Medical Sciences approved the study protocol and waived the need for written informed consent (2010—no. 90/122).

## Results

The characteristics of FSWs with and without a history of lifetime abortion are presented in Table 1. Overall, a total of 305 (35.3%, 95% CI: 32.1–38.6) reported a history of lifetime abortion. Assuming that FSWs in our study have had their first pregnancy at the age of 15, considering the mean age of 32, the average number of reproductive years was estimated to be roughly 17 years. Given the total reported number of 305 lifetime abortions, the annual rates of abortion among FSWs were estimated to be around 20.7 per 1000 FSWs [1000 × 305/ (32–15) × 863] = 20.7. Around 31.2% of FSWs (n = 251) reported no usual contraceptive use; however, barrier method, non-barrier modern contraception methods, and dual protection were reported by 32.6% (n = 262), 23.6% (n = 190), and 12.5% (n = 152) of the participants, respectively.

**Table 1:** Lifetime self-reported abortion in female sex workers with different socio-demographics, sexual behaviour, and injection risk behaviours in Iran (2010).

Variable	N	% Ever Aborted	P-value
		(95%CI)	
**Overall**	863	35.3 (32.1–38.6)	
**Age (years)**			
18–24	208	26.9 (21.0–33.5)	0.001
25–34	358	34.1 (29.2–39.2)	
≥35	297	42.8 (37.1–48.6)	
**Educational level**			
Never attended school	126	38.1 (29.6–47.2)	0.860
≤Primary school	258	33.7 (28.0–39.8)	
Guidance school	231	35.9 (29.7–42.5)	
≥High school	248	35.1 (29.2–41.4)	
**Ever married**			
No	161	25.5 (18.9–32.9)	0.004
Yes	702	37.6 (34.0–41.3)	
**Age at first marriage (years)**			
≤18	537	38.2 (34.0–42.4)	0.575
>18	165	35.8 (28.5–43.6)	
**Other sources of income than sex work**			
No	545	34.7 (30.7–38.8)	0.451
Yes	295	37.3 (31.8–43.1)	
**Have children**			
No	544	32.7 (28.8–36.8)	0.026
Yes	313	40.3 (34.8–45.9)	
**Length of sex work (years)**			
≤5	420	30.7 (26.4–35.2)	0.003
>5	443	40.2 (35.5–45.1)	
**HIV sero-status**			
Negative	779	34.7 (31.1–38.1)	0.600
Positive	30	30.0 (14.7–49.4)	
**Age at first sex (years)**			
≤18	645	37.5 (33.8–41.4)	0.021
>18	218	29.0 (23.0–35.4)	
**Age at sex work debut (years)**			
≤18	181	32.6 (25.8–39.9)	0.242
>18	613	37.4 (33.5–41.3)	
**Multiple paying partners (last working day)**			
No	517	31.5 (27.5–35.7)	0.004
Yes	346	41.0 (35.8–46.4)	
**Consistent condom use (paying partner)**			
No	309	32.0 (26.9–37.6)	0.065
Yes	523	37.5 (33.3–41.8)	
**Consistent condom use (non-paying partner)**			
No	171	40.3 (32.9–48.1)	0.403
Yes	290	41.4 (35.7–47.3)	
**Ever worked in brothels**			
No	541	31.2 (27.4–35.3)	<0.001
Yes	298	43.3 (37.6–49.1)	
**Group sex (last week)**			
No	751	33.6 (30.2–37.1)	<0.001
Yes	87	52.9 (41.9–63.7)	
**Ever traveled for sex work**			
No	533	30.0 (26.2–34.1)	<0.001
Yes	314	45.2 (39.6–50.9)	
**Sexual violence (last month)**			
No	576	26.9 (23.3–30.7)	0.001
Yes	287	46.0 (40.1–51.9)	
**STIs (last year)**			
No	410	27.6 (23.3–32.2)	<0.001
Yes	453	42.4 (37.8–47.1)	
**Accessed family planning services**			
No	397	27.7 (23.4–32.4)	<0.001
Yes	464	42.0 (37.5–46.7)	
**Self-perceived risk of HIV**			
No	482	31.3 (27.2–35.7)	0.006
Yes	381	40.4 (35.5–45.5)	
**Ever tested for HIV**			
No	453	30.9 (26.7–35.4)	0.004
Yes	409	40.3 (35.6–45.3)	
**Usual contraceptive use**			
Barrier	262	32.0 (26.7–37.6)	0.645
Non-barrier	190	22.7 (18.0–27.8)	
Dual protection	101	18.7 (14.4–23.5)	
None	251	26.7 (21.7–32.0)	
**Ever consumed alcohol**			
No	400	30.8 (26.3–35.5)	0.008
Yes	461	39.5 (35.0–44.1)	
**Ever used drugs**			
No	245	29.8 (24.1–35.9)	0.031
Yes	617	37.6 (33.8–41.6)	
**Ever injected drugs**			
No	737	33.1 (29.7–36.6)	0.001
Yes	125	48.8 (39.8–57.9)	

Higher prevalence of lifetime abortion was reported among FSWs who were older (42.8% vs. 26.9%; P-value = 0.001), had ever got married (37.6% vs. 25.5%; P-value = 0.004), had children (40.3% vs. 32.7%; P-value = 0.026), had been involved in sex work for longer (40.2% vs. 30.7%; P-value = 0.003), and were younger at first sex (37.5% vs. 29.0%; P-value = 0.021). Lifetime abortion was also higher among FSWs that had ever worked in brothels (43.3% vs. 31.2%; P-value<0.001), practiced group sex within past week (52.9% vs. 33.6%; P-value<0.001), ever travelled for sex work (45.2% vs. 30.0%), experienced sexual violence in the past month (46.0% vs. 26.9%; P-value = 0.001), reported STIs in the last year (42.4% vs. 27.6%; P-value<0.001), accessed family planning services (42.0% vs. 27.7%; P-value<0.001), self-perceived risk of HIV (40.4% vs. 31.3%; P-value = 0.006), and ever tested for HIV (40.3% vs. 30.9%; P-value = 0.004). Lastly, FSWs who had ever consumed alcohol (39.5% vs. 30.8%; P-value = 0.008), ever used drugs (37.6% vs. 29.8%; P-value = 0.031), and ever injected drugs (48.8% vs. 33.1%; P-value = 0.001) were more likely to have a history of lifetime abortion.

The results of the bivariable and multivariable analyses are presented in Table 2. In the multivariable logistic regression model, being older than 34 years (AOR = 1.74, 95% CI: 1.02, 2.96), recent group sex (AOR = 1.92, 95% CI: 1.10, 3.35), history of travel for sex work (AOR = 1.55, 95% CI: 1.09, 2.20), history of sexual violence (AOR = 1.77, 95% CI: 1.25, 2.50), reported STIs in last year (AOR = 1.53, 95% CI: 1.09, 2.14), and accessing family planning services (AOR = 1.76, 95% CI: 1.24, 2.49) remained independently significantly associated with history of lifetime abortion.

**Table 2:** Correlates of having a recent HIV test results among female sex workers in the first national bio-behavioural surveillance survey in Iran (2010).

Variables	Crude OR (95%CI)	P-value	Adj. OR*(95%CI)	P-value
**Age (years)**				
25–34 vs. 18–24	1.40 (0.96–2.00)	0.078	1.25 (0.79–1.97)	0.334
35≥ vs. 18–24	2.00 (1.40–3.00)	<0.001	1.74 (1.02–2.96)	0.039
**Ever married**				
Yes vs. No	1.77 (1.20–2.60)	0.004	1.53 (0.94–2.52)	0.084
**Have children**				
Yes vs. No	1.40 (1.04–1.80)	0.027	1.04 (0.74–1.47)	0.790
**Length of sex work**				
>5 vs. ≤5	1.50 (1.20–2.00)	0.003	1.21 (0.85–1.72)	0.283
**Age at first sex (years)**				
>18 vs. ≤ 18	0.68 (0.48–0.94)	0.021	0.92 (0.62–1.40)	0.670
**Multiple paying partners (last working day)**				
Yes vs. No	1.50 (1.10–2.10)	0.005	1.40 (0.98–1.90)	0.063
**Condom use with last paying partner**				
No vs. Yes	1.30 (0.94–1.70)	0.065	0.93 (0.59–1.50)	0.738
**Ever worked in brothels**				
Yes vs. No	1.70 (1.30–2.30)	0.001	1.14 (0.79–1.63)	0.459
**Group sex (last week)**				
Yes vs. No	2.20 (1.40–3.50)	<0.001	1.92 (1.10–3.35)	0.020
**Ever traveled for sex work**				
Yes vs. No	1.90 (1.40–2.60)	<0.001	1.55 (1.09–2.20)	0.014
**Sexual violence (last month)**				
Yes vs. No	2.00 (1.50–2.70)	0.001	1.77 (1.25–2.50)	0.001
**STIs (last year)**				
Yes vs. No	1.90 (1.50–2.60)	<0.001	1.53 (1.09–2.14)	0.013
**Accessed family planning services**				
Yes vs. No	1.90 (1.40–2.50)	<0.001	1.76 (1.24–2.49)	0.001
**Self-perceived risk of HIV**				
Yes vs. No	1.50 (1.12–1.97)	0.006	0.98 (0.69–1.40)	0.914
**Ever tested for HIV**				
Yes vs. No	1.50 (1.10–2.00)	0.004	1.21 (0.87–1.68)	0.251
**Ever consumed alcohol**				
Yes vs. No	1.50 (1.10–1.90)	0.008	1.10 (0.76–1.60)	0.584
**Ever used drugs**				
Yes vs. No	1.40 (1.00–1.90)	0.031	1.24 (0.83–1.86)	0.282
**Have ever injected drugs**				
Yes vs. No	1.90 (1.30–2.80)	0.001	1.25 (0.79–1.97)	0.326

*Variables with a P-value less than 0.2 in the bivariable analysis were entered into multivariable analysis. Data is not shown for variables with a P-value greater than 0.2 in the bivariable analysis.

## Discussion

Our study, the first of its kind in Iran, suggests that over 70% of FSWs in our national study were usually on contraceptives, and more than one-third had a history of lifetime abortion. Lifetime abortion was significantly associated with being older, as well as history of group sex within last week, travel for sex, sexual violence in last month, STIs in last year, and accessing family planning services. Our findings indicate that similar to a variety of international settings which have reported pregnancy termination prevalence ranging from 11.7% in Swaziland to over 80% in Cote d'Ivoire [14–20], FSWs’ reproductive health need are still unmet in Iran.

Moreover, FSWs’ contraceptive practices are comparable with those in women of reproductive age in the general population. While annual abortion rates among the general population are estimated 7.5 per 1000 women [10, 11], our findings suggest an estimate of about three times higher among FSWs. Although over two-third of Iranian FSWs and the general population (70.5% vs. 74.5%) have reported using a method of contraception, condom use practices of FSWs exceed that in the general population (30.6% vs. 11.4%). Conversely, women of the general population have reported higher practices of non-barrier modern contraception methods compared to FSWs (34.8% vs. 22.2%) [21]. Such differences between FSWs and women of the general population in Iran could be due to FSWs’ overstatement of condom use as well as higher fees associated with non-barrier modern contraception methods, active condom promotion efforts (e.g., free condom dispensation) catered towards FSWs receiving services at centers for vulnerable women, and higher self-perceived risk of FSWs for acquiring HIV/STIs [22–25].

Studies among FSWs have associated non-barrier modern contraception methods with reduced condom use, reflecting on a perceived decreased need for using barrier methods (i.e. condom) [18, 26, 27]. Among our participants, rates of condom use with last paying and non-paying partners were around 60% which is comparable with studies in various international settings [14–20]. Moreover, dual protection was reported among around 12.5% of our participants which is similar to studies elsewhere (e.g., 12% in Russia [18], 38% in Kenya [25]). Despite relatively high knowledge of HIV among Iranian FSWs and rising rates of condom use [12, 28], their inconsistent condom use could be influenced by barriers to condom use such as limited condom negotiation skills. This might urge FSWs to forgo condom use when on non-barrier modern contraception methods, despite their acknowledgment of the primacy of HIV risks associated with sex work [18]. Therefore, regardless of FSWs’ use of a non-barrier modern contraception method, the importance of consistent and correct condom use with all their sexual partners (paying and non-paying) should be further emphasized to prevent HIV/STIs transmission within their sexual networks. While promoting dual protection to FSWs may raise concerns around FSWs lower tendency towards using condoms while on other contraceptive methods [29], there is a great need for exploring alternative non-daily use or non-coitally dependent contraceptive methods (e.g., injectables and implants) on top of condoms among Iranian FSWs to reduce the risk of unintended pregnancies when they cannot follow correct and consistent condom use practices [25]. Nonetheless, promoting condom use with non-paying partners remains challenging in this sub-population as it may be interpreted as a sign of mistrust by their partners [20, 30, 31]. Despite the difficulties in accessing FSWs’ clients for health promoters in Iran, it is critical to find strategies (e.g., phone-based or web-based surveys) to direct prevention activities at paying and non-paying partners of FSWs.

History of travel for sex, group sex, and reported sexual violence were significantly associated with higher likelihood of lifetime abortion among FSWs. While our understanding of the mobility of FSWs in Iran is limited, this association is also reported in Afghanistan [22] and may reflect the importance of future mixed-methods research on the dynamics of selling sex outside the city of residence. Traveling for sex work purposes is associated with lower availability of contraception methods, higher risky sexual practices (e.g., violent sexual practices, anal sex), limited condom negotiation ability, and involvement in risky environments which often lead to harmful behaviours [22, 32, 33]. In particular, group sex -an indicator of high-risk behaviours- could be a frequent practice in such risky environments and has been associated with injection and non-injection drug use, selling sex, sex under the influence of drugs and alcohol, sexual mixing of HIV/STIs-infected people with non-infected individuals, and using sex toys and condoms with several sex partners [34, 35]. Our findings indicate the need to highlight the significance of contraceptive use and safe sex negotiation for FSWs, particularly among those who report traveling for sex or engaging in group sex events.

As expected, older age was significantly associated with a history of lifetime abortion as older FSWs’ desire for bearing children may decrease as they get older. Our finding, however, should not overshadow the importance of providing early access to contraceptives among young and adolescent FSWs as about one-fourth of 18-24-year-old FSWs in our sample reported a lifetime abortion. Indeed, studies suggest that given the illegal nature of abortion in several settings (e.g., Iran), the profound stigma associated with premarital pregnancy and sexual activity, limited sexual health education for youth and adolescents, and negative attitudes of healthcare providers towards teenage pregnancy, young women and FSWs’ abortion practices are highly under-reported and often carried out within unauthorized and underground health facilities [36–38].

Accessing family planning services—one of the main reasons for FSWs’ referrals to harm reduction services [24]–and self-reported STIs in the previous year—an indicator of high-risk behaviour—were significantly associated with higher lifetime abortion prevalence. This could be partly explained by the reverse causality effect of prior lifetime abortion experiences on current contraceptive use as well as the availability of STIs counseling services within these centers. While it is encouraging that despite Iran’s recent shift in population control policies, family planning services targeting FSWs are not significantly affected and are still freely available [39, 40], around 40% of our participants had not received family planning services (e.g., STIs counselling, free condoms). This calls for further research on barriers to accessing the available services.

We acknowledge the limitations of our study that are common to studies conducted on such sensitive topics among FSWs. Given that these data were collected in 2010, our findings may not give a full picture of current contraceptive and abortion practices of Iranian FSWs; however, we were not able to pursue publication earlier due to the sensitivities around the topic. Given the lack data on Iranian FSWs’ abortion practices, we believe our findings of the only national survey of FSWs to date, will still have crucial implications for both research and policy. Moreover, our cross-sectional study design limits causal inferences and the voluntary nature of participation and self-reported data do not rule out possible selection or social desirability biases. An important limitation of the study was the potential for time lag bias as the timing of abortion was not captured in the survey. However, given the difficulties around changing behaviour in any population including FSWs, those with prior abortions are likely to be exposed to present or future high risks of abortions. Our non-random sample may also limit the generalizability of our findings to high socioeconomic FSWs and those who do/cannot use the facilities serving FSWs (e.g., outreach FSWs). They might be likely to have different abortion experiences and seek abortion services in the private sector (e.g., high socio-economic FSWs) or underground (e.g., outreach FSWs). Nonetheless, whereas accessing a very representative sample of FSWs in Iran continues to be challenging, efforts were made to reduce potential biases by recruiting a large national sample size as well as engaging local organizations and staff as well as experienced interviewers.

### Conclusions

Our findings suggest that despite the recent efforts to improve the healthcare services available for FSWs, the reproductive health needs of Iranian FSWs are unmet and call for further research on the dynamics of FSWs’ contraceptive and abortion practices. Considering the criminalization around sex work and abortion in conservative settings such as Iran, providing safe abortion services for FSWs is a real and significant challenge; however, it is highly recommended to come up with strategies to provide abortion and post-abortion services to this vulnerable subpopulation. Future mixed-methods studies should examine the underlying reasons for non-use or discontinuation of contraceptive methods, decision-making process of seeking an abortion, dynamics of health facilities that offer abortion services, and barriers to accessing family planning services. As only 12.5% of the participants practiced dual protection, programs and policies should continue to highlight that next to pharmacotherapeutic approaches (e.g., antiretroviral treatment as prevention) only barrier methods could prevent HIV/STIs transmission. Harm reduction and family planning services should become more visible and accessible to FSWs, so they are equipped with the knowledge and services required to protect themselves against unwanted pregnancies and HIV/STIs risks. As the focus of current services targeting FSWs is HIV-related harm reduction, these efforts should also be expanded to consider their reproductive health of this marginalized population. Given the complexity of meeting the diverse and interconnected reproductive health needs of FSWs, efforts to address HIV, STIs, as well as unintended pregnancy would be most effective when made within an integrated reproductive health framework.
